# Ecophysiological response of *Populus alba* L. to multiple stress factors during the revitalisation of coal fly ash lagoons at different stages of weathering

**DOI:** 10.3389/fpls.2023.1337700

**Published:** 2024-01-10

**Authors:** Olga Kostić, Snežana Jarić, Dragana Pavlović, Marija Matić, Natalija Radulović, Miroslava Mitrović, Pavle Pavlović

**Affiliations:** Department of Ecology, Institute for Biological Research ‘Siniša Stanković’ - National Institute of the Republic of Serbia, University of Belgrade, Belgrade, Serbia

**Keywords:** *Populus alba*, fly ash, revitalisation, multiple abiotic stresses, potentially toxic elements, ecophysiological response

## Abstract

The enormous quantities of fly ash (FA) produced by thermal power plants is a global problem and safe, sustainable approaches to reduce the amount and its toxic effects are still being sought. Vegetation cover comprising long-living species can help reduce FA dump-related environmental health issues. However, the synergistic effect of multiple abiotic factors, like drought, low organic matter content, a deficit of essential nutrients, alkaline pH, and phytotoxicity due to high potentially toxic element (PTE) and soluble salt content, limits the number of species that can grow under such stressful conditions. Thus, we hypothesised that *Populus alba* L., which spontaneously colonised two FA disposal lagoons at the ‘Nikola Tesla A’ thermal power plant (Obrenovac, Serbia) 3 years (L3) and 11 years (L11) ago, has high restoration potential thanks to its stress tolerance. We analysed the basic physical and chemical properties of FA at different weathering stages, while the ecophysiological response of *P. alba* to multiple stresses was determined through biological indicators [the bioconcentration factor (BCF) and translocation factor (TF) for PTEs (As, B, Cr, Cu, Mn, Ni, Se, and Zn)] and by measuring the following parameters: photosynthetic efficiency and chlorophyll concentration, non-enzymatic antioxidant defence (carotenoids, anthocyanins, and phenols), oxidative stress (malondialdehyde (MDA) concentrations), and total antioxidant capacity (IC50) to neutralise DPPH free radical activity. Unlike at L3, toxic As, B, and Zn concentrations in leaves induced oxidative stress in *P. alba* at L11, shown by the higher MDA levels, lower vitality, and reduced synthesis of chlorophyll, carotenoids, and total antioxidant activity, suggesting its stress tolerance decreases with long-term exposure to adverse abiotic factors. Although *P. alba* is a fast-growing species with good metal accumulation ability and high stress tolerance, it has poor stabilisation potential for substrates with high As and B concentrations, making it highly unsuitable for revitalising such habitats.

## 1 Introduction

Fly ash (FA), the main by-product of coal-fired thermal power plants is a complex, heterogeneous material whose highly variable physical and chemical properties and toxicity are determined by the coal’s geological origin, the combustion process, the disposal method, the time the ash has been exposed to atmospheric conditions (age of the ash), and vegetation development (Haynes, 2009; Izquierdo and Querol, 2012; Bhatt et al., 2019; Kostić et al., 2022a). The intensification of activities related to this type of electricity generation is one of the major environmental problems today because the disposal of FA in dry or wet lagoons near thermal power plants contributes to the leaching of potentially toxic elements (PTEs) into soil and groundwater, while windblown micrometre-sized and poorly aggregated particles of FA from the dry surfaces of landfills pollute agricultural land and endanger the flora, fauna, and health of residents of nearby settlements (Raja et al., 2015; Ćujić et al., 2016; Khan and Umar, 2019).The formation of a vegetation cover, i.e. the revitalisation of FA landfills, is a cost-effective and environmentally sound method and the best method when it comes to stabilising this mobile substrate physically and chemically (Haynes, 2009; Yan et al., 2020; Kostić et al., 2022b). However, the synergistic effect of multiple abiotic stress factors, such as drought, low organic matter content, lack of essential nutrients (N, P, Mn, etc.), alkaline pH, and phytotoxicity due to high PTE and soluble salt content, limits the number of species that can grow under such conditions (Kostic et al., 2018; Kalashnikova et al., 2021). Due to its dark grey colour, FA absorbs enormous amounts of heat from the sun, which increases surface temperatures at the landfill and reduces humidity. This is further decreased by the ash’s sandy texture, which reduces water and nutrient retention capacity (Carlson and Adriano, 1991; Pavlović et al., 2004). Increased salinity, which can be as high as 13 dS m^-1^ in raw FA, can have a similar effect to drought on plants growing in landfills (Carlson and Adriano, 1993). When coal is burned, carbon (C) and nitrogen (N) are oxidised and transition to the gas phase, meaning FA is poor in both (Carlson and Adriano, 1993; Haynes, 2009). During and after combustion, the mineral fraction of coal undergoes various transformations that make the chemical elements bound in the original coal matrix susceptible to leaching during transport and disposal in landfills, especially when in contact with water (Izquierdo and Querol, 2012). In addition to the aluminosilicate matrix containing Si, Al, Fe, Ca, Mg, Na and K, the chemical composition of FA includes numerous microelements (As, B, Cu, Cr, Cd, Mn, Ni, Pb, Co, Mo, Zn, Se, etc.) whose concentrations in FA can be 4-10 times higher compared to the parent coal (Tian et al., 2018). Some of these elements are essential for plants in minimal amounts, while their presence in high concentrations can be potentially toxic. The most susceptible to leaching, and therefore most accessible to plants, are the partially volatile chemical elements (As, B, Cr, Se, and Zn) that condense in the surface layer of ash particles as the flue gases cool during the coal combustion process, while the less volatile elements such as Mn and Ni accumulate in the inner layer and are not directly exposed to leaching (Tian et al., 2018). The alkalinity of FA can cause the formation of insoluble forms of elements and thus a deficit of essential nutrients, most commonly phosphorus (P), manganese (Mn), and copper (Cu), but also increased solubility of arsenic (As), boron (B), and selenium (Se) and their accumulation in plants in toxic concentrations (Pandey et al., 2011; Gajić et al., 2020; Kostic et al., 2022c).

Abiotic stress factors lead to an overproduction of reactive oxygen species (ROS). These toxic products of incomplete oxygen reduction, which are common by-products of regular aerobic metabolic processes, are most commonly formed in chloroplasts, mitochondria, and cytoplasm, but also in membrane-bound exocellular enzymes involved in redox reactions, especially during photosynthetic electron transport and respiration (Sharma et al., 2020; Sachdev et al., 2021). Abiotic stresses such as high temperatures, drought, high salinity, and heavy metal pollution, as well as the combination of these stresses, disrupt the metabolic balance of cells, leading to increased production of ROS (Qadir et al., 2021; Mishra et al., 2023). In plants, this causes oxidative damage that disrupts many physiological processes, including photosynthesis as the main process of plant metabolism (Pavlović et al., 2007; Mitrović et al., 2012; Huang et al., 2019; Muhammad et al., 2021; Della Maggiora et al., 2023). Despite all the physical and chemical limitations and toxicity of FA, various plant species have been found growing on it (Kostić et al., 2012; Técher et al., 2012; Mukhopadhyay et al., 2017; Yadav et al., 2022). Thanks to the development of adaptation mechanisms based on accumulation and exclusion, tolerant species survive under conditions of PTE deficiency or toxicity in the substrate. By regulating the transfer between soil and roots and between roots and leaves, concentrations of these elements are kept within a normal range in their tissue (Pandey et al., 2012; Mukhopadhyay et al., 2017; Kostić et al., 2022b). Tolerant plant species also respond to abiotic stress by increasing their antioxidant capacity, which helps them to maintain a normal cellular balance between production and binding, degradation, and neutralisation of ROS by enzymatic or non-enzymatic antioxidants (carotenoids and anthocyanins) and secondary metabolites (phenols) (Cervilla et al., 2012; Gajić et al., 2020; Kebert et al., 2022; Vuksanović et al., 2022). Intolerant species exibit decreased antioxidant capacity and fewer mechanisms for detoxification in relation to ROS production, resulting in chain reactions in which free radicals damage important biomolecules, such as chloroplast pigments, lipids, and nucleic acids, oxidatively. Inactivation of enzymes and disruption of membrane structure follows, which leads to tissue damage and finally cell death (Moreno-Jiménez et al., 2009; Love et al., 2013; Stojnić et al., 2016; Gajić et al., 2020; Pilipović et al., 2020). In this sense, choosing the right plant species is an important factor in determining the efficiency of the FA landfill revitalisation process.

Previous studies have shown that autochthonous, long-living woody species characterised by high below- and above-ground biomass and rapid growth, as well as the ability to reproduce vegetatively and capacity to tolerate various stressors, are best suited to stabilising FA landfills and reducing health problems associated with FA while restoring ecosystem functions (Carlson and Adriano, 1991; Kostić et al., 2012; Mitrović et al., 2012; Pietrzykowski et al., 2018; Kostić et al., 2022b). Species from the *Salicaceae* family stand out when it comes to being used for the phytoremediation and revitalisation of polluted habitats, and in particular those from the *Populus* genus, which is characterised by high tolerance to a variety of environments (Zacchini et al., 2009; Guerra et al., 2011). However, species of *Populus*, as a thoroughly mapped genus, differ widely in their uptake patterns, as well as adaptation abilities under varying abiotic conditions (Dos Santos Utmazian et al., 2007; Zacchini et al., 2009; Di Lonardo et al., 2011; Tőzsér et al., 2023). To date, research has mostly focused on accumulation, phytoremediation potential, and tolerance of poplars to metal pollution (Madejón et al., 2004; Robinson et al., 2005; Borghi et al., 2008; Todeschini et al., 2011; Kostić et al., 2022b) or their physiological response to individual stress factors such as drought, salinity or high temperatures (Chen and Polle, 2010; Yoon et al., 2014; Zhang et al., 2019; Kim et al., 2022). However, studies on the ecophysiological response of *Populus alba* L. to the combined effects of multiple stress factors are rare, particularly at FA landfills. Therefore, the aim of this study is to contribute to the expansion of knowledge on the ecophysiological response of *P. alba* to single and combined multiple abiotic stresses at FA landfills and to test its suitability for the potential revitalisation of such habitats. We hypothesised that *P. alba*, which spontaneously colonised two FA disposal lagoons at the ‘Nikola Tesla A’ thermal power plant (TENT A), Obrenovac, Serbia, 3 years ago (L3) and 11 years ago (L11), has high restoration potential thanks to its stress tolerance. The first research objective was to evaluate some of the abiotic stress factors in FA lagoons at different stages of weathering by analysing (1) basic physical and chemical properties and (2) PTE (As, B, Cr, Cu, Mn, Ni, Se, and Zn) concentrations. The second objective was to assess the ecophysiological response of *P. alba* to abiotic stresses based on (1) determining its ability to regulate PTE transfer from soil to root and root to leaf and (2) its photosynthetic efficiency parameters (Fv/Fm), photosynthetic pigment content, non-enzymatic antioxidant defence parameters (carotenoids, anthocyanins, and phenols), oxidative stress parameters by measuring the concentration of malondialdehyde (MDA), and the total antioxidant capacity (IC50) for neutralizing the activity of DPPH free radicals. The results of this study, which would determine the temporal dynamics of the ecophysiological response of *P. alba* to single and combined abiotic stresses, could be of importance both for its application in the planned revitalisation of the TENT A landfill and for restoring similar landfills around the world.

## 2 Materials and methods

### 2.1 Description of the study sites

**Figure 1:**
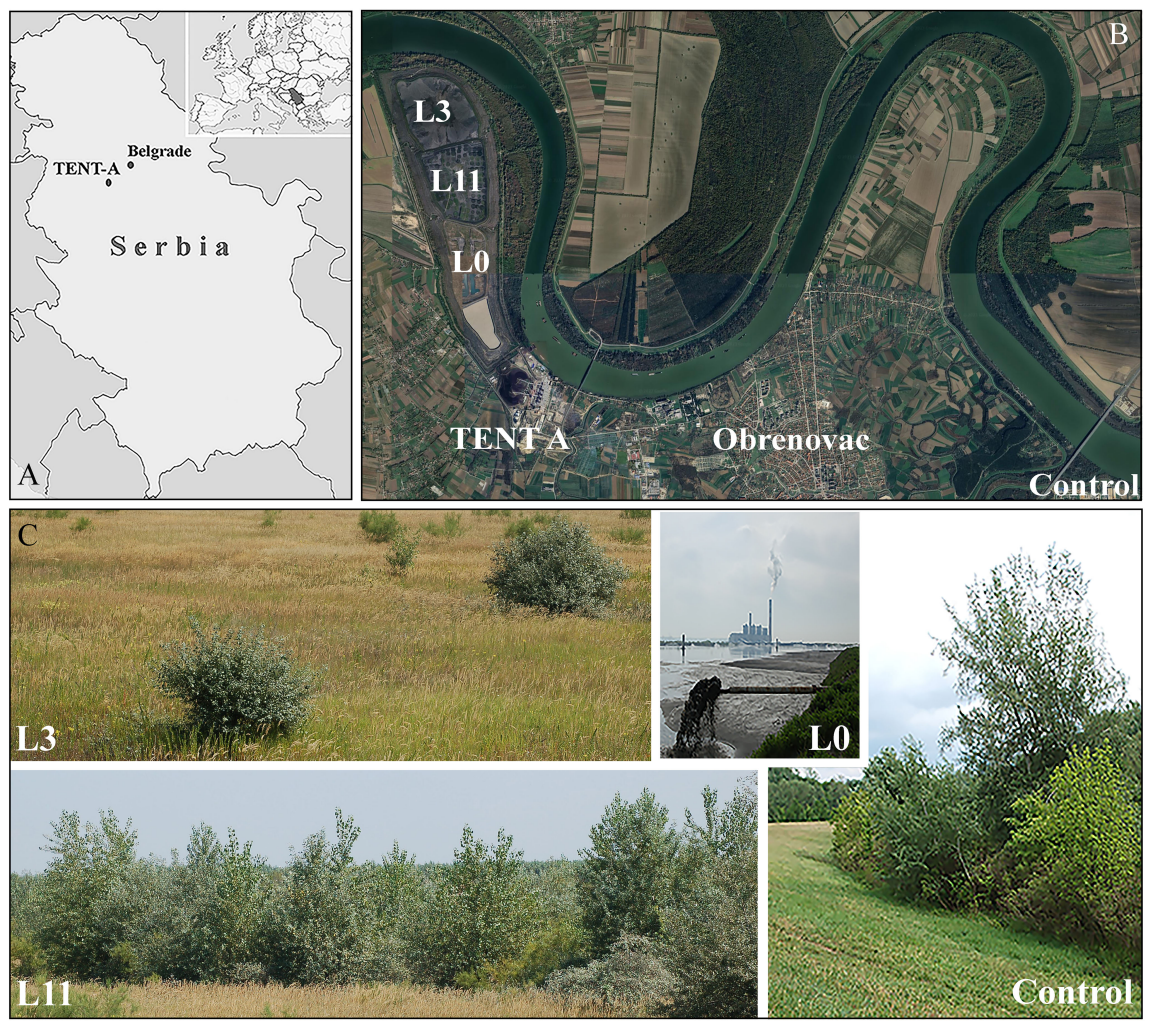
Study sites: **(A)** Geographical location of ‘Nikola Tesla A’ thermal power plant (TENT A) (images obtained from https://d-maps.com/carte.php?num_car=27552&lang=es; https://sh.wikipedia.org/wiki/Spisak_pozivnih_brojeva_u_Srbiji#/media/Datoteka:Serbia_in_Europe.svg, accessed on 21 July 2023), **(B)** Location of TENT A landfill (satellite image is obtained from GoogleEarth (https://earth.google.com/web/@44.69764779,20.15472413,76.16877517a,15536.9216476d,35y,0h,0t,0r/data=OgMKATA, accessed on 20 July 2023), **(C)** active lagoon (L0), and study sites at lagoons L3 and L11 and the natural habitat of *P. alba* on the bank of the Kolubara River (Control).

The TENT A FA landfill (44°40’19”N, 20°09’18”E) occupies 382 ha of the riparian section of the Sava River in the Belgrade municipality of Obrenovac, Serbia (**Figure 1**). This region is mainly characterised by a moderate-continental climate with warm summers and cold winters. The mean annual temperature is 12°C and total annual precipitation is 530 mm. During the year, periods of drought most often occur in August and September and are accompanied by extremely high temperatures (e.g. maximum temperatures in the period from July to August range from 38 to 42°C) (https://www.meteoblue.com/sr/vreme/historyclimate/weatherarchive/%D0%9E%D0%B1%D1%80%D0%B5%D0%BD%D0%BE%D0%B2%D0%B0%D1%86_%D0%A1%D1%80%D0%B1%D0%B8%D1%98%D0%B0_787516). In addition to data obtained from weather stations, we took measurements 5 cm above the surface of the ash during field research, which revealed temperatures ranging from 42.5°C in May to as high as 56°C in August.

The TENT A landfill is divided into three lagoons, one of which is always active in cycles of 6-12 years and into which 520 tonnes of ash and slag mixed with water (1:10) are hydraulically discharged every hour. After the two inactive lagoons have dried out, a grass-legume mixture is sown (*Arrhenatherum elatius* (L.) P. Beauv., *Dactylis glomerata* L., *Festuca rubra* L., *Lolium multiflorum* Lam., *Lotus corniculatus* L., *Medicago sativ*a L., *Secale cereale* L., and *Vicia villosa* Roth.; 270-300 kg ha^-1^) directly onto the ash without an insulating soil layer in order to prevent the dispersal of FA. Until the vegetative cover forms, each lagoon is also irrigated and fertilised (800 kg ha-1 of 15N:15P:15K) (Kostic et al., 2018). The study was conducted on the two inactive lagoons, L3 (three years after the start of revitalisation) and L11 (eleven years after the start of revitalisation), which at the time of the study had been spontaneously colonised by 99 species (55 at L3 and 80 at L11), 91% of which were herbaceous and 9% woody (4 at L3 and 9 at L11). Of the woody species present at both lagoons, *P. alba* was only sporadically present at L3, while it covered 30% of the area of L11 (Kostic et al., 2018). Control individuals of *P. alba* were selected in its natural habitat on the bank of the Kolubara River (Control), which is 10 km away from the TENT A FA landfill (**Figure 1**).

**Figure 2:**
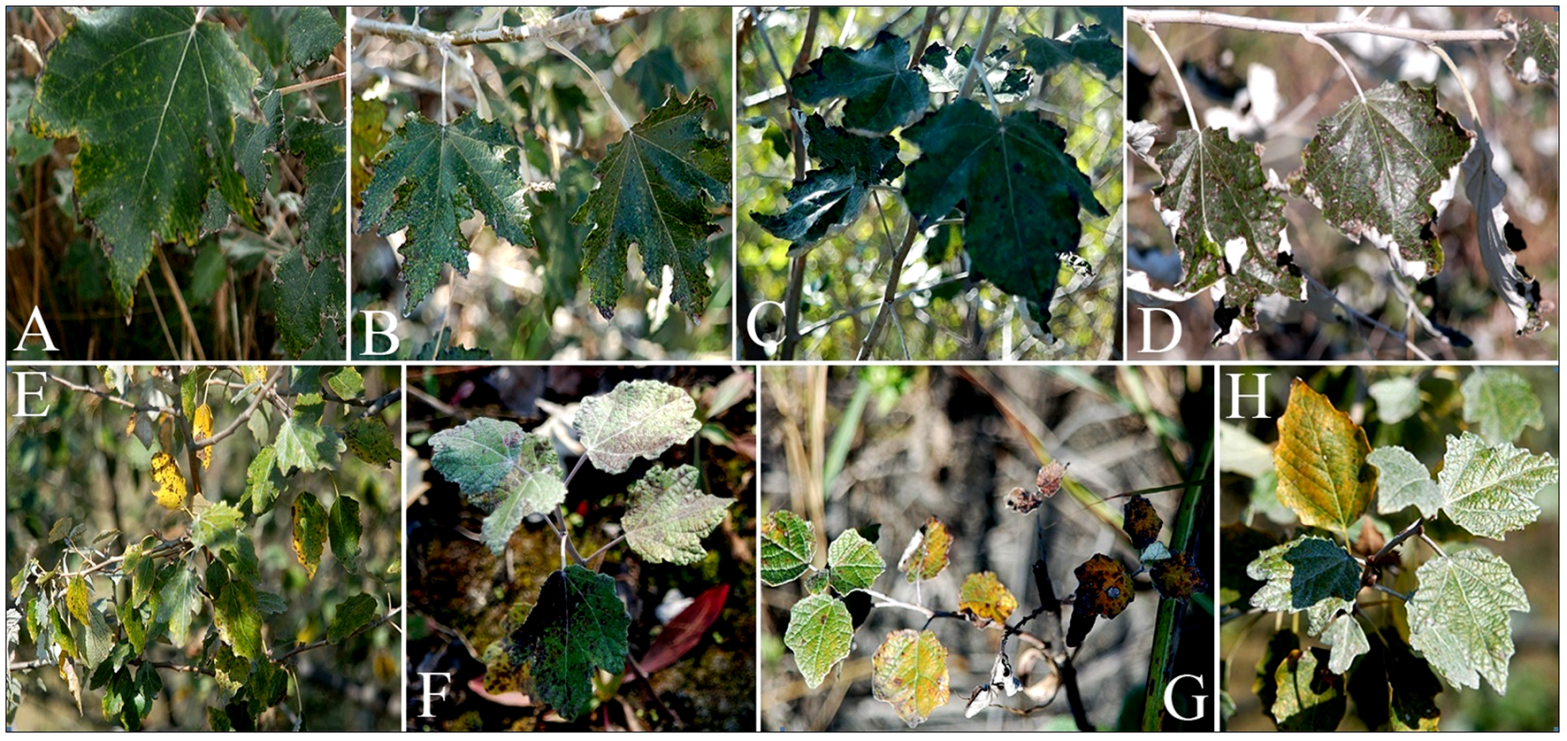
P*. alba* leaf damage symptoms at L3 **(A–D)** and L11 **(E–H)**.

During analysis of the leaves of the examined individuals, small necrotic spots were observed on leaves from L3 (**Figures 2A–D**), as well as chlorosis and marginal necrosis. At L11 (**Figures 2E–H**), the leaves were visibly smaller and thicker and leaf damage symptoms in the form of chlorosis and reddish-brown necrosis affecting a larger area of the leaf’s surface were also observed, while some leaves had completely dried out.

### 2.2 Plant species description

*P. alba* is a deciduous, anemophilous, mesophilous, and heliophilous woody species that tolerates frost and a wide range of different soil types and disturbed habitats, meaning it emerges spontaneously and grows rapidly under a variety of challenging conditions such as wildfires, clearings, and clearcuts. Because of its good adaptability to wet environments, it is most common on floodplains near rivers, lakes, and canals (Palancean et al., 2018). It naturally grows on all floodplains along major rivers in eastern, central, and southwestern Europe, as well as in southwestern and central Asia, but especially on the floodplains of the Danube River and its tributaries (Caudullo et al., 2017). Thanks to its wide ecological range, it is naturalised worldwide. *P. alba* is propagated by seeds, but also vegetatively thanks to its widely branched surface veins and great sprouting capacity. Due to its distinctive root system and tolerance to salt and sandy soil, it is used to strengthen and stabilise coastal dunes by preventing them from being washed away during floods (Caudullo and de Rigo, 2016).

### 2.3 Sample collection and preparation

Samples of rhizosphere FA and soil (at a depth of 0-30 cm) and plant material (roots and leaves of *P. alba*) were collected from five sampling plots (15 x 15 m) and from randomly selected specimens at each lagoon at approximately equal distance from the lagoon edge (25-30 m) and at the control site. Leaf samples were collected from the same height and from all four cardinal points. FA and soil samples (250 g per sample) were dried at room temperature and then sieved through a 2-mm aperture sieve before being quartered into representative samples (~500 g) for each site. For the analysis of PTE concentrations, the leaf and root samples for each site were combined, washed, and then dried at 65°C to constant weight and ground in a laboratory mill (Polymix, Kinematica AG, 2 mm aperture mesh, stainless steel sieve). The leaf samples for the analysis of the biochemical parameters were stored at -80°C until further analysis.

### 2.4 Analysis of physical and chemical characteristics of fly ash and control soil

pH (WTW - Germany, inoLab 7110 pH meter) and electrical conductivity (EC µS.cm^-1^, Knick, Germany, Portamess 911 conductivity meter) in FA and soil samples were determined in a 1:5 suspension of FA (soil) to distilled water. Total organic carbon (C%) was determined using Simakov’s modified Turin method (Simakov, 1957) and total nitrogen (N%) was determined using the semi-micro Kjeldahl method. Particle size distribution was carried out using a combined pipette and sieve technique with a 0.4 N sodium pyrophosphate solution, while fractionation was performed according to Atterberg (Atterberg, 1911). Based on the sand, silt and clay content, FA and soil texture was determined using the International Soil Texture Triangle (Soil Survey Division Staff, 2017). The C/N ratio was calculated using Equation (1), as the ratio of the average content of total organic carbon (C) to the average content of total nitrogen (N) in FA and soil, for each of the three study sites.


(1) $$C/N=\frac{\text{C}}{\text{N}}$$

### 2.5 Analysis of concentrations of potentially toxic elements in fly ash, soil, and plant samples

Inductively coupled plasma optical emission spectrometry (ICP-OES, Spectro Genesis, Spectro-Analytical Instruments GmbH, Kleve, Germany) was used to determine PTE concentrations (mg kg^-1^) in FA, soil, and plant (root and leaf) samples. Prior to analysis, samples were subjected to wet digestion in a microwave oven (CEM, Mars 6 Microwave Acceleration Reaction System, Matthews, NC, USA). The US EPA acid digestion method 3051 [U.S. Environmental Protection Agency (USEPA), 1998] was used to determine the pseudo total concentrations of PTEs in FA and soil samples, i.e. their maximum content that can be available to plants, as this method does not ensure complete digestion of the elements bound to silicate mineral fraction. The total content of PTEs in root and leaf samples was obtained by means of US EPA acid digestion method 3052 [U.S. Environmental Protection Agency (USEPA), 1996]. The detection limits for the analysed PTEs were as follows: As - 0.005, B - 0.005, Cr - 0.001, Cu - 0.001, Mn - 0.001, Ni - 0.009, Se - 0.007, and Zn - 0.005. To verify the accuracy of the analytical procedures, certified reference materials were analysed: FA (coal ash BCR - 038), soil (clay ERM - CC141) and plant material (beech leaves BCR - 100) provided by IRMM (Institute for Reference Materials and Measurements, Geel, Belgium) and certified by EC-JRC (European Commission - Joint Research Centre).

### 2.6 Measurements of photosynthetic efficiency and pigment content

The maximum quantum yield of Photosystem II (PSII), or photosynthetic efficiency (Fv/Fm), was measured under field conditions using a portable fluorometer (Plant Stress Meter, BioMonitor SCI AB, Sweden) and calculated as Fm - Fo/Fm (Krause and Weis, 1991). Fo (minimum fluorescence level) was measured once *P. alba* leaves had been dark adapted for 30 minutes, while Fm (maximum fluorescence level) was measured after chlorophyll had been excited by a 2-second pulse of saturated actinic light with a density of 200-400 µmol photons m^-2^s^-1^. Photosynthetic pigments such as chlorophyll a (Chl a), chlorophyll b (Chl b), and total carotenoids (Tot Carot) were extracted from *P. alba* leaves with 80% acetone. The absorbance of the supernatant was measured at 663 nm, 645 nm, and 480 nm using a spectrophotometer (UV-Vis spectrophotometer, Shimadzu UV-160) and the chlorophyll and carotenoid content was calculated (Arnon, 1949; Wellburn, 1994) and expressed as mg g^-1^ dry weight. Total chlorophyll content (Chl a + b), their ratio (Chl a/b), and the chlorophyll to carotenoid ratio (Chl a + b/Tot Carot) were calculated.

### 2.7 Analysis of oxidative stress parameters and total antioxidant protection

The content of malondialdehyde (MDA; nmol g^-1^ of fresh weight) in leaf samples of *P. alba* (0.5 g) was determined after homogenisation in 5 mL of 80% ethanol with 0.05 mL of 2% butylated hydroxytoluene (Heath and Packer, 1968). A solution of 1 mL of the supernatant, 0.5 mL of 0.65% thiobarbituric acid, and 0.5 mL of 10% trichloroacetic acid was heated to a temperature of 95°C for 15 minutesbefore being cooled on ice and centrifuged at 3,000 g for 10 minutes. The absorbance of the supernatant was measured using spectrophotometry at 450 nm, 532 nm, and 600 nm. Anthocyanin (Anthoc; mg g^-1^ of dry weight) in the leaves was extracted with 1 ml DMSO at 65°C for 2 hours and then heated at 65°C for a further 4 hours after the addition of 0.5 ml 2N HCl. The absorbance of the supernatant was measured spectrometrically at 650 nm, 620 nm, and 520 nm (Creasy, 1968; Proctor, 1974). Free phenols (Ph Free; mg g^-1^ of dry weight) in the leaves were extracted with 80% (v/v) boiling aqueous methanol solution followed by ethyl acetate. Bound phenols (Ph Bound; mg g^-1^ of dry weight) were extracted by boiling the insoluble residue of the Ph Free extraction in 2N HCl for 60 min and then it was transferred to ethyl acetate (Djurdjevic et al., 2007). Absorbance was measured spectrophotometrically at 660 nm (Feldman and Hanks, 1968) and a standard curve was constructed using different concentrations of ferulic acid (Serva, Heidelberg, Germany). The total antioxidant capacity of *P. alba* leaves was determined using free radical DPPH (1,1-diphenyl-2-picrylhydrazyl) (Brand-Williams et al., 1995). 0.5 g of leaves was homogenised in 10 mL of 95% ethanol and samples were prepared at three increasing extract concentrations (5 µL, 25 µL, and 50 µL) with the addition of 0.5 mL DPPH. Absorbance (A) was measured using spestrophometry at 517 nm. DPPH radical scavenging ability (%) was calculated as ((Acontrol - Asample)/Acontrol) x 100. The ‘effective concentration’ or IC50 (mg ml^-1^), which indicates the amount of plant extract that results in DPPH activity decreasing by 50% (Molyneux, 2004), was used to determine the total antioxidant activity of *P. alba* at all the sites studied. A lower value points to higher antioxidant activity.

### 2.8 Statistical analysis

**Figure 3:**
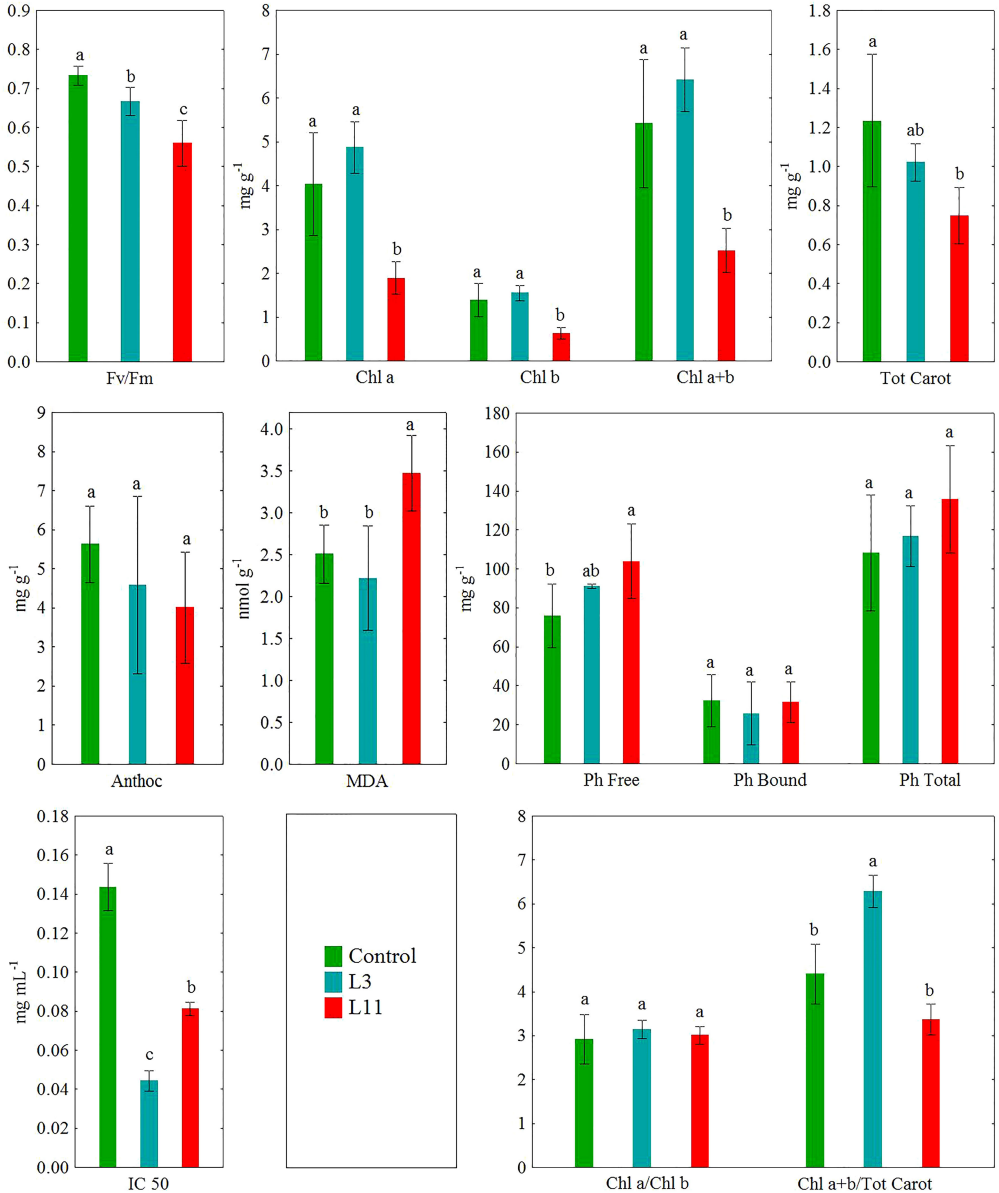
The ecophysiological response of the leaves of *P. alba* on fly ash (L3 and L11) and soil (Control). Different letters indicate significant difference between sites at P<0.001.

**Figure 4:**
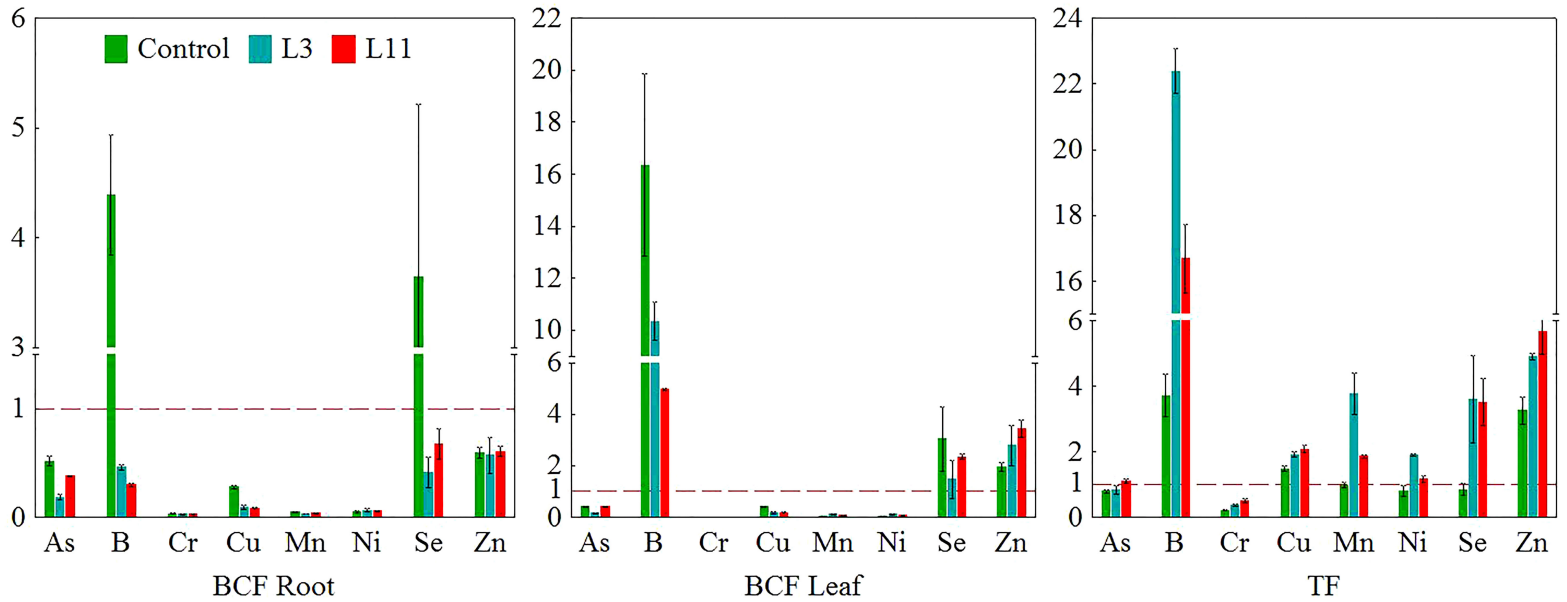
Bioconcentration factors for PTEs in roots (BCF Root) and leaves (BCF Leaf) and translocation factors (TF) for *P. alba* on fly ash (L3 and L11) and soil (Control).


The comparison of means (M) with standard deviation (SD) of 5 replicates (n=5) between the three investigated sites for each analysed parameter is shown in **Tables 1**–**3** and **Figure 3**. The obtained data was analysed using statistical analysis (ANOVA) and means were separated with a Bonferroni test at a level of significance of P<0.001, using the Statistica software package (Weiß, 2007). Data was checked to ensure it met the assumptions for ANOVA before it was analysed. The efficiency of *P. alba* to remove or stabilise chemical elements in FA (**Figure 4**) was assessed according to biological indicators such as the bioconcentration factor (BCF), calculated using Equation (2), and the translocation factor (TF), calculated using Equation (3), (Zacchini et al., 2009; Zhenggang et al., 2023).

**Table 1:** Physical and chemical properties of fly ash at lagoons L3 and L11 and soil (Control).

	pH_H2O_	EC µS cm^-1^	C %	N %	C/N	Sand 2.0-0.02 mm	Silt + Clay <0.02 mm
L3							
Mean	7.95 **a**	242.60 **a**	2.01 **b**	0.05 **c**	45.14	83.13 **a**	16.87 **c**
SD	0.08	8.14	0.25	0.01		1.34	0.60
L11							
Mean	7.70 **b**	201.80 **b**	1.43 **c**	0.12 **b**	12.35	71.80 **b**	28.20 **b**
SD	0.01	939	0.05	0.01		1.48	1.48
Control							
Mean	7.55 **c**	138.40 **c**	4.08 **a**	0.38 **a**	10.74	8.27 **c**	91.73 **a**
SD	0.02	5.59	0.12	0.04		0.93	0.93

(One-way ANOVA - Bonferroni); Data represents Mean values with standard deviation (SD) of five replicates (n=5); Means followed by the same letter in a column do not differ significantly between sites (P<0.001).

**Table 2:** Pseudo total concentrations of PTEs (mg kg^-1^) in fly ash at lagoons L3 and L11 and soil (Control).

	As	B	Cr	Cu	Mn	Ni	Se	Zn
L3								
Min	23.70	47.64	76.84	41.12	198.84	41.50	0.97	32.93
Max	32.08	52.10	107.56	57.00	254.59	61.53	2.25	45.13
Mean	**27.52a**	**47.64a**	**86.00a**	48.12 **a**	218.22 **b**	49.42 **b**	1.68 **a**	39.45 **a**
SD	3.05	2.65	12.65	5.87	21.38	7.62	0.51	5.49
L11								
Min	13.72	41.16	52.65	38.55	205.36	49.36	1.27	31.97
Max	14.88	42.00	67.18	45.52	227.05	58.41	1.80	37.46
Mean	14.23 **b**	**41.16b**	58.10 **b**	42.96 **a**	213.13 **b**	53.15 **b**	1.49 **a**	35.48 **a**
SD	0.46	0.61	5.70	2.87	9.13	3.72	2.22	2.20
Control								
Min	8.00	2.21	55.43	15.69	430.29	68.59	0.14	39.30
Max	8.36	3.00	69.10	17.46	466.67	69.64	0.30	46.34
Mean	8.22 **c**	2.61 **c**	62.76 **ab**	16.46 **b**	443.90 **a**	68.70 **a**	0.23 **b**	42.11 **a**
SD	0.14	0.31	5.00	0.66	14.32	1.11	0.08	2.62
Threshold and average concentrations in soils								
*Average range ^a^*	4.4-8.4	22-40	47-51	13-23	270-525	13-26	0.25-0.34	45-60
*Critical* *for plants ^b^*	20-50	25 ^c^	75-100	60-125	1500-3000	100	5-10	70-400
*Background* *Kolubara River ^d^*	10.3		67.5	22.24		54.87		69
*Average* *Central Serbia ^e^*	11		48	27		58		48
*Average World ^e^*	10		50	30		20		50

(One-way ANOVA - Bonferroni); data represents minimum (min), maximum (max), and Mean values with standard deviation (SD) of five replicates (n=5); Means followed by the same letter in a column do not differ significantly between sites (P<0.001); Mean critical concentrations are in bold. Threshold and average concentrations in soil: ^a^ Kabata-Pendias and Pendias, 2001; ^b^ Alloway, 1990; ^c^ Kloke et al., 1984; ^d^ Čakmak et al., 2018; ^e^ Mrvić et al., 2009.

**Table 3:** PTE concentrations (mg kg^-1^) in the roots (Root) and leaves (Leaf) of *P. alba* at the study sites.

Root	As	B	Cr	Cu	Mn	Ni	Se	Zn
L3								
Min	4.86	21.16	2.17	4.24	6.35	2.99	0.50	20.93
Max	5.36	22.89	2.38	4.48	8.13	3.12	1.00	23.39
Mean	**5.04 a**	21.99 **a**	2.27 **a**	4.32 **a**	6.79 **b**	3.02 **a**	0.68 **a**	22.16 **a**
SD	0.21	0.73	0.11	0.11	0.76	0.06	0.21	0.89
L11								
Min	4.84	11.72	1.37	3.37	7.48	3.12	0.75	19.84
Max	5.72	13.02	1.61	3.97	8.06	3.23	1.37	23.84
Mean	**5.33a**	12.29 **b**	1.49 **b**	3.66 **b**	7.81 **b**	3.16 **a**	1.01 **a**	21.58 **a**
SD	0.33	0.54	0.09	0.22	0.22	0.06	0.22	1.59
Control								
Min	3.98	9.61	1.75	4.35	19.14	3.00	0.50	22.09
Max	4.87	12.11	2.50	4.88	22.97	3.75	1.12	27.01
Mean	4.47 **a**	11.41 **b**	2.15 **a**	4.62 **a**	20.80 **a**	3.49 **a**	0.82 **a**	25.19 **a**
SD	0.36	1.04	0.33	0.26	1.51	0.36	0.23	1.91
Leaf	As	B	Cr	Cu	Mn	Ni	Se	Zn
L3								
Min	3.50	481.76	0.75	8.10	24.40	5.62	2.00	104.17
Max	4.63	505.63	0.87	8.39	26.28	5.88	2.75	113.80
Mean	4.24 **b**	**491.97a**	0.85 **a**	8.29 **a**	25.49 **a**	5.73 **a**	2.33 **b**	**108.71b**
SD	0.47	9.51	0.05	0.13	0.96	0.11	0.27	3.66
L11								
Min	5.60	201.49	0.74	7.31	13.95	3.49	2.99	119.38
Max	6.23	208.49	0.75	8.12	15.23	3.87	4.24	123.30
Mean	**5.83 a**	**204.94b**	0.75 **a**	7.59 **a**	14.57 **c**	3.72 **b**	3.49 **a**	**122.00a**
SD	0.25	2.68	0.00	0.31	0.53	0.21	0.50	1.59
Control								
Min	3.23	41.21	0.37	6.58	19.63	2.73	0.37	79.91
Max	3.88	42.87	0.50	7.08	21.10	2.75	0.87	83.31
Mean	3.52 **b**	42.18 **c**	0.45 **b**	6.78 **b**	20.32 **b**	2.74 **c**	0.70 **c**	81.78 **c**
SD	0.23	0.68	0.07	0.21	0.56	0.01	0.21	1.27
Threshold and average concentrations in plants								
**Deficit**	–	3-30	–	2-5	10-30	–	–	10-20
**Normal**	1-1.7	10-100	0.01-0.5	5-30	30-300	0.1-5	0.01-2	27-150
**Toxic**	5-20	50-200	5-30	20-100	400-1000	10-100	5-30	100-400

(One-way ANOVA - Bonferroni); data represents minimum (min), maximum (max), and Mean values with standard deviation (SD) of five replicates (n=5); Means followed by the same letter in a column do not differ significantly between sites (P<0.001, 2); Mean toxic concentrations are in bold. Threshold and average concentrations in plants: ^a^ Kabata-Pendias and Pendias, 2001.

(2) $$BCF=\frac{C_{Root\;\left(Leaf\right)}}{C_{FA\left(Soil\right)}}$$

(3) $$TF=\frac{C_{Leaf}}{C_{Root}}$$

**Figure 5:**
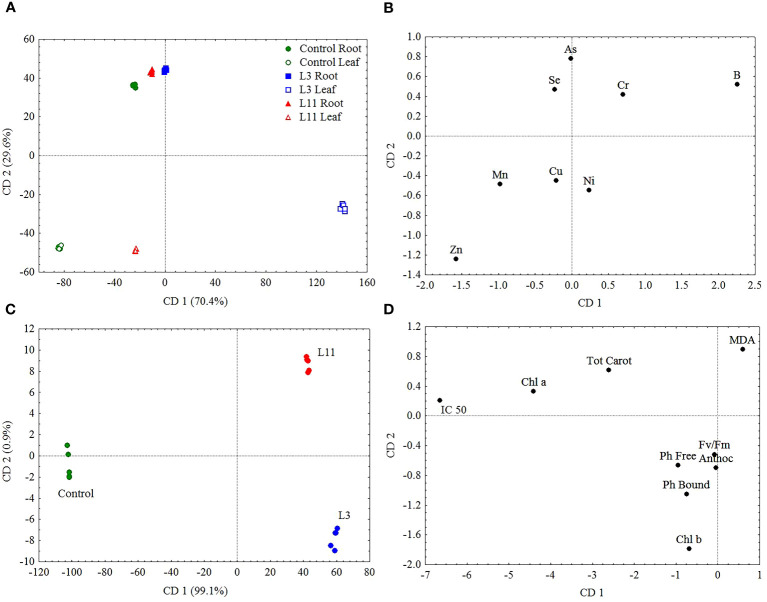
Canonical discriminant analysis (CDA) based on PTE concentrations in roots and leaves **(A)** and **(B)** and ecophysiological response parameters **(C)** and **(D)** of *P. alba* at L3, L11, and the Control site.

To determine the link and intensity of the effect of PTE concentrations in leaves on the parameters of the ecophysiological response of *P. alba* non-parametric Spearman rank-order correlation was used at a level of significance of P<0.001 (**Table 4**). Canonical discriminant analysis (CDA) was conducted to detect which parameter had the greatest impact on the differences established between the plants from the study sites (**Figure 5**).

**Table 4:** Spearman’s correlations between ecophysiological parameters and PTE concentrations in *P. alba* leaves across all the examined sites.

Parameters	As	B	Mn	Zn
**Fv/Fm**	**-0.981**	-0.218	0.634	**-0.931**
**Chl a**	**-0.822**	0.410	**0.960**	-0.544
**Chl b**	**-0.872**	0.309	**0.931**	-0.629
**Tot Carot**	**-0.937**	-0.256	0.562	**-0.892**
**Anthoc**	-0.502	-0.350	0.210	-0.609
**Ph Free**	**0.891**	0.344	-0.457	**0.878**
**Ph Bound**	0.298	-0.441	-0.450	-0.019
**MDA**	**0.845**	-0.349	**-0.926**	0.586
**IC 50**	-0.405	**-0.951**	-0.343	-0.745

Bold indicates significant correlation at P<0.001.

## 3 Results

### 3.1 Basic physical and chemical characteristics of fly ash and soil

The basic physical and chemical properties of FA from lagoons L3 and L11 and soil from the natural habitat of *P. alba* (Control), sampled in the rhizosphere layer (at a depth of 0-30 cm), are presented in **Table 1**.

Compared to the Control soil, FA from L3 and L11 was characterised by a higher pH and EC and a higher proportion of the Sand fraction (L3>L11>Control; P<0.001), but lower C (Control>L3>L11; P<0.001) and N content (Control>L11>L3; P<0.001) and a lower proportion of the Silt+Clay fraction (Control>L11 >L3; P<0.001). In addition, FA from L3 had a higher pH, EC, C, C/N, and sand fraction content (P<0.001), but lower N and Silt+Clay fraction content (P<0.001) compared to FA from L11 (**Table 1**). In terms of soil classification (Soil Survey Division Staff, 2017), the control soil was classified as silty clay loam soil, while the particle size distribution of FA revealed that the texture of FA at L3 was similar to that of loamy sand and FA from L11 similar to that of sandy loam.

### 3.2 Concentrations of potentially toxic elements in fly ash and soil

Pseudo total concentrations of PTEs (As, B, Cr, Cu, Mn, Ni, Se, and Zn) in FA from lagoons L3 and L11 and soil (Control) are shown in **Table 2**.

Significantly higher concentrations (P<0.001) of As (235%), B (1725%), Cu (192%), and Se (630%) were found at L3, as well as As (73%), B (1477%), Cu (161%), and Se (548%) at L11, compared to those in soil. Lower concentrations of Mn (51-52%) and Ni (29-23%) were found in both lagoons compared to the Control, where the content of Cr and Zn was similar to the content in FA. Concentrations of PTEs in the Control soil generally did not exceed the average values for world soils and those in central Serbia (Mrvić et al., 2009), as well as the background values for the Kolubara Basin (Čakmak et al., 2018). The exception was slightly elevated concentrations of Cr and Ni due to soil formation processes on basic and ultrabasic rocks, which have a high natural content of these elements (Geological Information System of Serbia - GEOLIS, 2010; Pavlović et al., 2021). At L3, concentrations of As (93%), B (16%), and Cr (48%), which were in the critical range for plants (Kloke et al., 1984; Alloway, 1990), were higher than at L11, where B was still in the critical range while As and Cr were in a range higher than the average for world soils (Kabata-Pendias and Pendias, 2001; Mrvić et al., 2009). Similar concentrations of Cu, Ni, and Se, which were in a range higher than the average for world soils, and Mn and Zn, which were below the average, were determined in FA from both lagoons (**Table 2**).

### 3.3 Potentially toxic elements in roots and leaves of *P. alba*

Concentrations of PTEs in *P. alba* roots and leaves at the study sites are shown in **Table 3**. The biological indicators BCF and TF for the examined elements in *P. alba* are shown in **Figure 4**.

Concentrations of As, Ni, Se, and Zn measured in the roots of *P. alba* were similar at all three sites. However, B was higher at L3 than at the control site, while Cr and Cu were lower at L11 and Mn was lower at both lagoons compared to the control. Concentrations of B, Cr, Cu, Ni, Se, and Zn in the leaves of *P. alba* at both lagoons and of As at L11 were higher than in the control individuals, for which the content of most PTEs was in the normal range. The exceptions were As, whose content in *P. alba* leaves was higher than average values, and Mn, whose content was in the deficit range for plants (**Table 3**; Kabata-Pendias and Pendias, 2001). At L3, concentrations of B, Mn, and Ni in leaves were higher than at L11, while As, Se, and Zn levels were lower. Cr and Cu content was similar at both lagoons. Of all the elements tested, toxic concentrations of B and Zn were measured in *P. alba* leaves at L3 and of As, B, and Zn at L11, while Mn was in the deficit range at both lagoons. Cr, Se (L3 and L11), As and Ni (L3) levels in leaves were found to be in a range higher than normal but not toxic, while Cu was in the normal range at both lagoons (**Table 3**; Kabata-Pendias and Pendias, 2001). Analysis of BCF and TF showed that BCF in roots (BCF Root) was less than 1 for all PTEs studied at all the sites except for B and Se at the control site. BCF in leaves (BCF Leaf) was higher than 1 for B, Se, and Zn at all the sites. A TF higher than 1 was determined for B, Cu, Mn, and Zn at all three sites, for Ni and Se at L3 and L11, and for As only at L11. A general increase in the levels of the biological indicators above 1 (L3<L11) was found for As, Cu (TF), and Zn (BCF Leaf and TF) (**Figure 4**).

### 3.4 The ecophysiological response of *P. alba* to abiotic stresses at fly ash lagoons

The ecophysiological response of *P. alba*, based on Fv/Fm measurements and analysis of the content of metabolites such as Chl a, Chl b, Tot Carot, Anthoc, Ph Bound, Ph Free, MDA, and IC 50, is shown in **Figure 3**. The results of canonical discriminant analysis (CDA), which singles out those PTEs and ecophysiological parameters that are most responsible for the differences in the response of *P. alba* to stress factors, are shown in **Figure 5**. Spearman’s rank correlations between all the examined ecophysiological parameters and PTE concentrations in *P. alba* leaves deviating from the range of normal values at all the sites are shown in **Table 4**.

In *P. alba* leaves, significantly lower values (P<0.001) of Fv/Fm (10%) and IC 50 (71%) were determined at L3 and of Fv/Fm (24%), Chl a (53%), Chl b (12%), Chl a+b (54%), Tot Carot (39%), and IC 50 (43%) at L11 than at the control site. Compared to the control individuals, there was a higher Chl a+b/Tot Carot ratio (39%) at L3 and a higher content of Ph Free (37%) and MDA (39%) at L11. In addition, lower values of Fv/Fm (16%), Chl a (58%), Chl b (59%), and Chl a+b (61%) were found in *P. alba* leaves at L11 compared to L3, but higher MDA (57%). Statistically significant differences in the content of Anthoc, Ph Bound, and Ph Free, as well as in the Chl a/Chl b ratio were not determined at the study sites for *P. alba* leaves (**Figure 3**).

In terms of the variability in concentrations of the examined PTEs in roots and leaves and the ecophysiological response of *P. alba* at the examined sites, the results of canonical discriminant analysis (CDA) showed that As, B, and Zn (**Figures 5A, B**), as well as IC 50, Chl a, Chl b, Tot Carot, and MDA (**Figures 5C, D**), had the greatest impact on the differences between the study sites. Across all the examined sites, the content of As, Se, and Zn in *P. alba* leaves correlated negatively with Fv/Fm and the Tot Carot content, but positively with Ph Free content. There was a strong negative correlation between chlorophyl content and As and a positive one with Mn content in leaves. However, the association of MDA with these elements exhibited the opposite trend. The total antioxidant activity of *P. alba* correlated negatively with B content in leaves, while the content of Ph Bound and Anthoc had a weak association with the examined PTEs in *P. alba* leaves (**Table 4**).

## 4 Discussion

At FA landfills, numerous kinds of abiotic stress simultaneously and continuously affect plants as a result of the unfavourable physical and chemical properties of the ash, the extreme microclimatic conditions, and the deficit or toxicity of essential and non-essential chemical elements. Such conditions hinder the survival and development of vegetation because they threaten the normal course of physiological processes in plants, including photosynthesis, which is extremely sensitive to heat, drought, salinity, heavy metal toxicity, and nutrient deficiency or toxicity (Muhammad et al., 2021). Research has shown that a combination of multiple stressors has a cumulative effect on almost all the physiological parameters that result in increased plant damage (Pandey et al., 2015; Zandalinas et al., 2021). Many studies have shown that plants vary in terms of their ability to adapt and survive under conditions of multiple and varying stresses at ash landfills and that their survival is most often based on a strong functional response and their antioxidant capacity (Gajić et al., 2020; Kalashnikova et al., 2021; Qadir et al., 2021; Qadir et al., 2022). In addition, successful plants are able to immobilise PTEs in the root zone rather than accumulate them in leaves (Mitrović et al., 2008; Kostić et al., 2022b). Therefore, by analysing the functional properties of plants that survive in conditions of multiple stresses, it is possible to select species that are suitable for the processes of the revitalisation of polluted habitats and thus contribute to reducing pollution in the vicinity of large industrial complexes.

Analysis of chlorophyll fluorescence is a useful tool for studying the impact of abiotic stress on plants (Guidi et al., 2019). It provides information on the state of PSII, i.e. to what extent it uses the energy absorbed by chlorophyll and to what extent these stress conditions lead to damage to the photosynthetic apparatus. In this sense, calculating the photosynthetic efficiency parameter (Fv/Fm) is crucial for detecting the photoinhibition of PSII under stress conditions. The results of our study showed that at the control site there was good water availability due to occasional flooding, an adequate water table, and well-structured silty clay loam. The soil was also slightly alkaline (7.4-7.8; Soil Survey Division Staff, 2017), there was an adequate mineral supply, and concentrations of the studied PTEs in the leaves of the control plants were in a range below the critical values for plants (Kabata-Pendias and Pendias, 2001). This provided optimal conditions for the growth of *P. alba* as shown by Fv/Fm values, which were in the optimal range for plants (0.750-0.850; Bjorkman and Demmig, 1987). However, Fv/Fm values for *P. alba* leaves at the FA lagoons were significantly lower at L3 (ranging between 0.650-0.687), indicating photoinhibition of PSII and a decrease in *P. alba* vitality with a tendency to further decrease at L11 (ranging between 0.510-0.585) due to prolonged stress and the accumulation of photosynthetic tissue damage, visible through pronounced morphological symptoms in the form of necrosis and leaf drying. A decrease in photosynthetic efficiency has been found in many plants at FA landfills such as *Dactylis glomerata* L., *Ricinus communis* L., *Solanum lycopersicum* L., *Tamarix tetrandra* Pall. ex M. Bieb., *Pinus sylvestris* L., *Picea abies* (L.) H. Karst., *Fagus sylvatica* L., and *Alnus glutinosa* (L.) Gaertn. (Gajić et al., 2020; Bęś et al., 2021; Kostic et al., 2022c; Qadir et al., 2022).

The general reduction in photosynthetic efficiency in the examined species at both FA lagoons is the result of the influence of multiple stressors such as drought, heat, and salt, but also inadequate mineral nutrition (PTE toxicity or deficit). In general, drought, salt, and heavy metal stress have been found to have almost similar effects on plant growth as they result in the production of ROS and oxidative stress (Zhang et al., 2019; Krzyżak et al., 2023). Specifically, the weaker binding properties of FA particles due to their texture, which corresponds to the texture of loamy sand soils, and low organic matter content resulted in the ash having a lower water-retention capacity, which exposed *P. alba* to drought stress at L3. Despite the fact that the hydraulic transport of raw FA during its disposal at L3 caused a significant reduction in the ash’s salinity due to drainage of the transport water and the drying out of the FA (Kostic et al., 2018), the salt content in the ash at that lagoon was still significantly higher (242.60µS cm^-1^) than in the control soil. This has a drought-like effect on the plants and is reflected in the reduction in photosynthetic efficiency (Técher et al., 2012). It should be noted that at L11 both abiotic (weathering) and biotic (vegetation development) factors that inevitably accompany the FA disposal process at landfills contribute to the increase in the Silt+Clay fraction and the narrowing of the C/N ratio as the best indicator of organic matter accumulation. They also result in a reduction in pH and the content of soluble salts in FA due to their leaching, which should increase the capacity of the FA to retain water and nutrients (Uzarowicz et al., 2017; Konstantinov et al., 2020; Petrović et al., 2023). However, at the time of our research, maintenance of the vegetative cover at L11 was no longer carried out (i.e. it was not watered), unlike at L3, so there was a moisture deficit despite the changes in the physical and chemical characteristics. The moisture deficit at the TENT A landfills is further intensified by the excessive heating of the surface layers of FA due to its dark grey colour, which was confirmed by the extremely high temperatures that were measured on the surface of the FA lagoons in the summer months. Moreover, due to the pozzolanic properties of FA particles, their wetting in the presence of liming material (CaSO4) cements them together, which is why compacted layers, which reduce aeration, water infiltration, and root penetration, form in places (Carlson and Adriano, 1993). Although flexible plant roots can grow horizontally or develop through zones of least resistance such as root canals, root elongation and water infiltration is reduced by the hard, compact layers of FA (Haynes, 2009). Stomatal closure as a common response to drought reduces CO2 uptake and fixation, but also reduces transpiration, limiting the cooling of the leaf’s surface, resulting in thermal stress that has negative effects on metabolic processes, primarily photosynthesis. Thermal stress also causes changes in chlorophyll pigment and carotenoid content in leaves, thus affecting photoinhibition, which results in a reduced quantum yield of PSII (lower Fv/Fm), enhanced peroxidation in the leaf cell membrane (an increase in MDA), and reduced membrane thermostability (Yoon et al., 2014; Stojnić et al., 2016; Pilipović et al., 2020; Rosso et al., 2023). Increased salinity limits plant growth by inducing osmotic stress, impairing the uptake of ions leading to their imbalance or toxicity, inhibiting metabolic processes and enzyme activity, damaging thylakoid membranes, reducing photosynthetic pigments, and disrupting photosynthetic activity (Chen and Polle, 2010; Zhang et al., 2019).

Two of poplar’s main strategies to cope with drought stress are avoidance mechanisms that control transpiration through the regulation of stomatal conductance, the deposition of cuticular waxes to limit non-stomatal transpiration, increased root growth, and reduced leaf area (Attia et al., 2015) and a stress tolerance strategy based on mechanisms to maintain biological functions through the synthesis of protective molecules and compounds under stress conditions (Viger et al., 2016). Salt tolerant poplar species are characterised by restricted xylem translocation and salt accumulation in the roots (Chen and Polle, 2010). However, our research found a reduction in photosynthetic efficiency of *P. alba* at both FA lagoons, which confirms earlier findings concerning *P. alba x P. glandulosa* (Yoon et al., 2014) and *P. alba × P. davidiana* (Kim et al., 2022) on the sensitivity of poplar to drought and salt stress, which leads to changes in their metabolic activity.

In terms of PTE pollution, among the PTEs determined in the FA at L3, As, B, and Cr were the main contaminants with concentrations exceeding critical levels for plants (As>20 mg kg^-1^, B>25 mg kg^-1^, Cr>75 mg kg^-1^; Kloke et al., 1984; Kabata-Pendias and Pendias, 2001). Concentrations of Cu, Ni, and Se were higher than the average for sandy to silt-loam substrates and concentrations of Mn and Zn were lower than the range of average values (<270 mg kg^-1^ and<45 mg kg^-1^, respectively; Kabata-Pendias and Pendias, 2001). The surface association of As, B, Cr, and Zn on FA particles contributes to their greater solubility, making these elements extremely hazardous to plants at FA landfills if they are accumulated in high concentrations (Gajić et al., 2020; Kostić et al., 2022b; Kostic et al., 2022c). However, it also contributes to their significantly lower content in FA at L11, although the content of B at L11 was still in the critical range for plants. As essential elements, B and Zn are easily transported (BCF Leaf>1; TF>1) to leaves (Kabata-Pendias and Pendias, 2001; Robinson et al., 2005), while As and Cr as non-essential chemical elements mostly accumulate in roots (Wang et al., 2002; Singh et al., 2013). Under these conditions at the TENT A landfills, *P. alba* accumulated toxic concentrations of B and Zn (>50 mg kg^-1^, >100mg kg^-1^; Kabata-Pendias and Pendias, 2001) at L3 and L11 and also toxic concentrations of As (>5 mg kg^-1^; Kabata-Pendias and Pendias, 2001) at L11, while Mn content was in the deficit range (<30 mg kg^-1^; Kabata-Pendias and Pendias, 2001). The accumulation potential for B and Zn in *P. alba* leaves at L3 and L11 is also confirmed by earlier studies on poplars (Madejón et al., 2004; Robinson et al., 2005; Dos Santos Utmazian et al., 2007; Robinson et al., 2007; Di Lonardo et al., 2011; Rees et al., 2011; Huang et al., 2019; Pilipović et al., 2019; Kostić et al., 2022b; Tőzsér et al., 2023). The lower content of B in *P. alba* leaves at L11 compared to L3 was accompanied by changes in B content in FA because its solubility at pH>6 is conditioned only by its total concentration (Iwashita et al., 2005). The increase in Zn and As content in leaves at L11 compared to L3 is a result of their greater accumulation and translocation to leaves at this lagoon. This is due to the higher bioavailability of Zn and As, which is in line with the changed physical and chemical characteristics of FA at L11 compared to L3 – namely, a higher proportion of finer particles and organic matter, lower pH and salinity, and the absence of supplementation with phosphate fertilisers. This contributes to the greater solubility and mobility of the elements (Kostic et al., 2018; Balafrej et al., 2020; Gajić et al., 2020). Specifically, unlike at L3, where the application of phosphate fertilisers compensated for the phosphorus deficit in the FA, the cessation of phosphorus supplementation at a later stage of revitalisation and its leaching caused a deficit in the FA at L11, which can result in increased uptake of As by plants through the phosphate transport system (Wang et al., 2002). The poor solubility of Mn in FA is the cause of its characteristic deficit in plants growing at ash landfills (Adriano et al., 2002; Mitrović et al., 2008; Kostić et al., 2022b). Namely, earlier findings suggest that Mn content in *P. alba* leaves correlates to its availability in surface soil (Madejón et al., 2004) and that the accumulation potential of poplar for Mn is limited (Tőzsér et al., 2023). The higher uptake and transport of Zn through plant tissue to *P. alba* leaves at L11 may be caused by the lower Mn content in leaves resulting from its reduced bioavailability in FA at L11 due to the altered physical and chemical characteristics of FA when compared to L3 (Kabata-Pendias and Pendias, 2001; Aref, 2011). Cr content in *P. alba* leaves at L3 and L11 was higher than normal, but not toxic (>0.5 mg kg^-1^; Kabata-Pendias and Pendias, 2001). In our study, much higher Cr concentrations were measured in *P. alba* roots than in leaves, confirming earlier findings for poplar trees (Pulford and Watson, 2003; Ancona et al., 2017; Tőzsér et al., 2023). BCF Root<1 and TF<1 make *P. alba* suitable for the phytostabilisation of this element.

The results of discriminatory analysis showed that of all the analysed PTEs in *P. alba* parts at the study sites, toxic concentrations of As, B, and Zn and a deficit of Mn contributed most to differences in the response of *P. alba* to the effects of stress. Of these differences, changes in the content of Chl a, Chl b, Tot Carot, MDA, and IC 50 were the most pronounced (**Figure 5**). Specifically, the toxic content of B in leaves may inhibit photosynthesis and significantly reduce Fv/Fm by damaging thylakoid membranes and reducing CO2 assimilation. This, in turn, causes disruption to photosynthetic electron transport, oxidative damage, and decreased photosynthetic enzyme activity, as well as reduced synthesis of δ-aminolevulinic acid and protochlorophyll as a precursor of chlorophyll biosynthesis as noted by a large number of researchers (Ardic et al., 2009; Han et al., 2009; Wang et al., 2011; Cervilla et al., 2012; Chen et al., 2012; Landi et al., 2012). At L3, typical visible symptoms of B toxicity were noted in the form of chlorosis and tip and marginal leaf necrosis (Rees et al., 2011). Nevertheless, the fact that there are differences in the tolerance levels of different poplar species is shown by the hybrid species *Populus nigra × euramericana*. Only 10% of its leaves exhibited damage at concentrations of up to 900 mg B kg^−1^, while chlorosis occurred with concentrations of 1000-2000 mg B kg^−1^ and tissue necrosis only at concentrations greater than >2000 mg B kg^−1^ (Rees et al., 2011). Photosynthesis is the first metabolic process that will be disrupted by toxicity of Zn, which leads to a loss of plasma membrane integrity and a reduction in its permeability, damage to chloroplast functioning, and a reduction in photosynthetic electron transport (Huang et al., 2019; Balafrej et al., 2020). It has been found that high concentrations of Zn in poplar induce chlorotic and necrotic changes, alter leaf morphology and ultrastructure, leading to significant thickening of the leaf lamina and spongy tissue, and reduce above-ground biomass (Todeschini et al., 2011). It is possible that the extensive symptoms of damage in the form of chlorosis, necrosis, and leaf drying and also the visible smaller leaves with thicker lamina at L11 are the result of long-term toxic concentrations of Zn in poplar leaves. However, the toxic As levels measured in *P. alba* leaves at L11 can also adversely affect photosynthetic efficiency as it leads to disruption of the electronic transport chain, which conditions changes in the formation of NADPH and ATP and results in increased chlorophyll fluorescence (Stoeva and Bineva, 2003; Rahman et al., 2007; Gusman et al., 2013). This can be seen in our research through the decrease in Fv/Fm values, but also the visible morphological changes in the form of red-brown necrosis and the drying of older leaves (Kabata-Pendias and Pendias, 2001). Previous research has shown that *P. alba* accumulates higher amounts of As in leaves at contaminated sites than at uncontaminated ones (Madejón et al., 2004) and that the impact of As toxicity on chlorophyll synthesis is particularly pronounced (Moreno-Jiménez et al., 2009), which may be associated with the decrease in chlorophyll content at L11. Manganese is a chemical element that is directly involved in electron transport reactions and is essential for the synthesis of chlorophyll. Therefore, its deficit leads to the destruction of chloroplast thylakoid membrane structure, a reduction in the number of PSII reaction centres, and the degradation of complexes for the photolysis of water, in which four Mn atoms provide the energy necessary for the oxidation of two water molecules to oxygen (Alejandro et al., 2020).

In terms of photosynthetic pigments in leaves, measuring chlorophyll concentrations is an effective way to determine plants’ tolerance to stress. At L3, *P. alba* was found to have lower vitality, but significant differences in the content of Chl a, Chl b, Tot Carot, Anthoc, Ph Free, Ph Bound, and MDA compared to control individuals were not found. This may be the result firstly of NPK fertilisation, as N application improves the drought tolerance of poplars (Song et al., 2019), secondly of the higher Mn content in leaves compared to the other two sites as one of the mechanisms for overcoming stress (Della Maggiora et al., 2023), and thirdly the higher total antioxidant capacity of *P. alba* at this lagoon (<IC 50) as an indicator of the activation of enzymatic and non-enzymatic antioxidant systems (Zhang et al., 2019; Rosso et al., 2023). Also, the localisation of high concentrations of accumulated B in chlorotic and necrotic tissues in the tips and marginal parts of leaves is another adaptive mechanism of plants, which thus ensures a significant proportion of photosynthetic tissue remains functional (Rees et al., 2011). Unlike *P. alba* at L3 and the control site, a lower content of Chl a, Chl b, and Tot Carot and a higher content of MDA in the leaves along with a higher IC 50 was found at L11, indicating its less effective antioxidant defence system, which is consistent with the very low Fv/Fm values measured at that site. The significant correlation between As, Mn, and Zn in leaves and the analysed ecophysiological parameters (**Table 4**) indicates that the increase in As and Zn concentrations, as well as the decrease in Mn concentrations in leaves at L11 induces its weaker adaptive response at the older lagoon. The accumulation of both physiological and morphological damage indicates the importance of the long-term impact of stress on the process of photosynthesis in *P. alba* (Muhammad et al., 2021). Phenols as secondary metabolites contribute to physiological processes that are associated with protection against abiotic stress (Bencherif et al., 2020). As such, the higher Ph Free content in leaves at L11 when compared to the control site can be seen as the antioxidant response of *P. alba* to the environmental stresses at that particular site. However, it was not sufficient to quench lipid peroxidation and scavenge ROS under drought and salt stress conditions, as well as elevated PTE content. In particular, the toxic As and Zn concentrations contribute to this, as indicated by the positive correlation between Ph Free and the content of these two elements in leaves (**Table 4**). Also, the cytotoxic and prooxidative nature of phenolic compounds can be expressed in plants growing on FA due to the high content of Fe and Al oxides with the formation of phenoxy radicals, the life of which is significantly extended in the presence of Zn (Sakihama and Yamasaki, 2002). Through the synthesis of carotenoids and anthocyanins, plants also actively increase tolerance to different types of stress (Cervilla et al., 2012). However, unlike some species of plant in which a high As content in leaves induces an increased accumulation of anthocyanins (Leão et al., 2014), in woody species, such as *P. alba* in this case, and in earlier studies on *Tamarix tetrandra* at FA landfills, their antioxidant role is absent (Kostic et al., 2022c). The degradation in carotenoids for *P. alba* observed at L11 results in a reduction in the plant’s resistance to ROS, which favours a rise in lipid peroxidation rates and MDA concentrations, as was also found in *T. tetrandra*, *Cassia occidentalis, and Dactylis glomerata* at FA landfills (Love et al., 2013; Gajić et al., 2020; Kostić et al., 2022b). The exposure of *P. alba* to oxidative stress at L11 is also indicated by the decrease in the Chl a + b/Tot Carot ratio compared to L3, which occurs because of the accumulation of leaf tissue damage and progressive tissue aging due to its prolonged exposure to environmental stresses (Stoeva and Bineva, 2003). This is unlike the Chl a/b ratio as an early warning indicator of the toxic effects of metal accumulation in plants (D’Addazio et al., 2023), which remains unchanged compared to L3. These results are in line with earlier research that also showed a decrease in chlorophyll and carotenoid content, as well as an increase in MDA in poplars in response to drought (Kim et al., 2022) and heavy metal stress (Chandra and Kang, 2016). A decrease in chlorophylls and carotenoids has also been found in many plants at FA landfills, such as *T. tetrandra, Robinia pseudoacacia* L., *Amorpha fruticosa* L., and *Withania somnifera* (Pavlović et al., 2004; Qadir et al., 2021; Kostic et al., 2022c).

## 5 Conclusion

This study was conducted with the aim of evaluating the ecophysiological response of *Populus alba* L., which spontaneously colonised two FA disposal lagoons at the ‘Nikola Tesla A’ thermal power plant (Obrenovac, Serbia) 3 years (L3) and 11 years (L11) before the beginning of the research, to simultaneous and continuous exposure to abiotic stressors. It was found that at both lagoons *P. alba* was exposed to multiple stress factors such as drought and salt stress, but also inadequate PTE concentrations. At L3 there were critical concentrations of As, B, and Cr in FA and at L11 critical concentrations of B, while a deficit of Mn is characteristic for all plants growing on such substrates. The *P. alba* individuals at both lagoons showed lower vitality than the individuals in their natural habitat. In addition, the effects of single and combined stressors on *P. alba* trees resulted in visible leaf damage symptoms in the form of chlorosis, necrosis, and drying of leaves. Some of these symptoms are characteristic of drought, increased salinity, toxic concentrations of B and Zn, and deficient Mn in *P. alba* leaves and were more pronounced due to the increase in toxic concentrations of As at L11. Nevertheless, care measures (e.g. watering, fertilisation) and the increased biosynthesis of enzymatic and non-enzymatic antioxidant compounds (<IC 50) at the beginning of the revitalisation process contributed to the better response of *P. alba* to stress at L3, as indicated by the absence of differences in levels of Chl a, Chl b, Tot Carot, Anthoc, Ph Free, Ph Bound, and MDA compared to the control individuals. In contrast to L3, the increased toxic concentrations of As and Zn in leaves at L11 resulted in oxidative stress in *P. alba*, as evidenced by lower vitality, reduced synthesis of chlorophyll, carotenoids and total antioxidant activity, and higher MDA content. This suggests that despite its rapid colonisation of FA lagoons, the stress tolerance of *P. alba* decreases with increasing intensity and duration of exposure to adverse abiotic factors. Furthermore, its weaker stabilisation potential for substrates with high As and B content makes *P. alba* highly unsuitable for the permanent revitalisation of such habitats. Although white poplar is a fast-growing species with a good ability to accumulate metals and a high stress tolerance, the above characteristics could give it an advantage, but only if it were used during the temporary, short-term restoration process in the cyclical disposal of FA in the passive lagoons at the landfill and with the application of care measures such as ash moistening and NPK supplementation.

## Data availability statement

The raw data supporting the conclusions of this article will be made available by the authors, without undue reservation.

## Author contributions

OK: Conceptualization, Data curation, Investigation, Methodology, Writing – original draft, Writing – review & editing. SJ: Formal Analysis, Investigation, Validation, Writing – review & editing. DP: Formal Analysis, Validation, Writing – review & editing. MMa: Formal Analysis, Investigation, Writing – review & editing. NR: Formal Analysis, Investigation, Writing – original draft. MMi: Conceptualization, Methodology, Supervision, Writing – review & editing. PP: Funding acquisition, Supervision, Writing – review & editing.
